# Enhancing SAPS-3 Predictive Accuracy with Initial, Peak, and Last Lactate Measurements in Septic Shock

**DOI:** 10.3390/jcm13123505

**Published:** 2024-06-15

**Authors:** Arthur Stoiber, Alexander Hermann, Sophie-Theres Wanka, Gottfried Heinz, Walter S. Speidl, Christian Hengstenberg, Peter Schellongowski, Thomas Staudinger, Robert Zilberszac

**Affiliations:** 1 Department of Medicine I, Medical University of Vienna, 1090 Vienna, Austria; 2 Department of Cardiology, Medical University of Vienna, 1090 Vienna, Austria

**Keywords:** septic shock, lactate, lactate dynamics, ICU prognostication, SAPS-3, critical care outcomes

## Abstract

**Background/Objectives**: Septic shock is a severe condition with high mortality necessitating precise prognostic tools for improved patient outcomes. This study aimed to evaluate the collective predictive value of the Simplified Acute Physiology Score 3 (SAPS-3) and lactate measurements (initial, peak, last, and clearance rates within the first 24 h) in patients with septic shock. Specifically, it sought to determine how these markers enhance predictive accuracy for 28-day mortality beyond SAPS-3 alone. **Methods**: This retrospective cohort study analyzed data from 66 septic shock patients at two ICUs of Vienna General Hospital (2017–2019). SAPS-3 and lactate levels (initial, peak, last measurement within 24 h, and 24 h clearance) were obtained from electronic health records. Logistic regression models were constructed to identify predictors of 28-day mortality, and receiver operating characteristic (ROC) curves assessed predictive accuracy. **Results**: Among 66 patients, 36 (55%) died within 28 days. SAPS-3 scores significantly differed between survivors and non-survivors (76 vs. 85 points; *p* = 0.016). First, last, and peak lactate were significantly higher in non-survivors compared to survivors (all *p* < 0.001). The combination of SAPS-3 and first lactate produced the highest predictive accuracy (AUC = 80.6%). However, 24 h lactate clearance was not predictive of mortality. **Conclusions**: Integrating SAPS-3 with lactate measurements, particularly first lactate, improves predictive accuracy for 28-day mortality in septic shock patients. First lactate serves as an early, robust prognostic marker, providing crucial information for clinical decision-making and care prioritization. Further large-scale studies are needed to refine these predictive tools and validate their efficacy in guiding treatment strategies.

## 1. Introduction

Sepsis and its severe progression, septic shock, are critical conditions that pose significant challenges within intensive care units (ICUs) globally. Characterized by profound organ failure and a high mortality rate, which remains between 40 and 60% despite advances in medical treatments, septic shock necessitates precise and dynamic prognostic tools to effectively guide therapeutic decisions [1].

The Simplified Acute Physiology Score 3 (SAPS-3) is one such prognostic tool, designed to estimate the likelihood of hospital mortality based on data at ICU admission [2,3]. Among the disadvantages of the SAPS-3 is that it is assessed only once, thus precluding any evaluation of disease progression. The SAPS-3, not specifically designed for sepsis or septic shock, estimates only the mortality risk and not morbidity. It includes variables with considerable interpretive leeway, such as the Glasgow Coma Scale and factors related to pre-ICU conditions and patient history. Additionally, vital parameters recorded at admission may be skewed by immediate actions taken before assessment, such as repositioning and emergency treatments, potentially impacting score accuracy. Other scoring systems specifically developed for sepsis were often designed using small sample sizes and are not sufficiently validated [4]. SAPS-3 has the advantage of being developed across a diverse range of ICUs in 35 countries, with specific models tailored for various global regions, often using small samples. Typically, applying this score to populations outside the original study group results in reduced performance, primarily seen as poorer calibration, while discrimination generally remains stable [5].

This highlights an ongoing need to refine and adapt prognostic models to better suit global patient populations.

Lactate, as a biomarker, plays a pivotal role in the management of sepsis and septic shock. Elevated lactate levels are indicative of cellular hypoxia and metabolic stress, conditions prevalent in sepsis due to impaired tissue perfusion [6]. Recent evidence, however, challenges the traditional view that sepsis-associated hyperlactatemia primarily results from hypoxia and anaerobic metabolism [7].

Traditionally, a lactate level exceeding 2 mmol/L is considered a hallmark of septic shock and has been strongly linked with increased mortality rates [8]. However, lactate’s utility extends beyond a simple diagnostic threshold; dynamic changes in lactate levels, particularly lactate clearance over time, have been associated with patient outcomes, reflecting the effectiveness of resuscitative efforts [9].

Clinical studies highlight the importance of initial lactate levels and their clearance rate, with lactate clearance—defined as the percentage decrease over typically 24 h—being predictive of survival in septic shock. Rapid lactate clearance within the first 24 h of ICU admission is associated with better outcomes, indicating its potential as a real-time indicator of treatment response [10,11].

The integration of lactate measurements with SAPS-3 potentially improves its prognostic accuracy. This study sought to evaluate the additional value of initial, peak, last, and clearance rates of lactate alongside SAPS-3 for predicting 28-day mortality in septic shock patients. Through a comprehensive retrospective analysis, we investigated whether enhancing SAPS-3 with detailed lactate profiles offers better predictive performance than using SAPS-3 alone. Furthermore, given the complex interplay between lactate kinetics and patient survival [12,13,14], this study not only aimed to validate the integration of lactate metrics with SAPS-3 but also to dissect the temporal and qualitative aspects of lactate changes as prognostic enhancers in septic shock management. Such insights could fundamentally alter clinical protocols, emphasizing a more nuanced approach to the use of lactate in ICU settings.

## 2. Materials and Methods

### 2.1. Study Objectives

The primary objective of this retrospective cohort study was to assess the additive predictive value of incorporating lactate parameters with SAPS-3 in predicting 28-day mortality for patients diagnosed with septic shock. Specifically, we aimed to examine how initial lactate, peak lactate within 24 h, lactate after 24 h, and lactate clearance improve the predictive accuracy of SAPS-3 alone. The secondary objective was to explore the prognostic capabilities of these lactate parameters independently.

### 2.2. Study Design

We conducted a retrospective analysis of patient data from two intensive care units situated at the Vienna General Hospital from January 2017 to December 2019. The local ethics committee of the Medical University of Vienna approved the study (protocol code 1853/2019, last re-approved on 25 July 2023), and due to its retrospective design, the requirement for informed consent was waived.

### 2.3. Study Population

Patients eligible for this study were 18 years of age or older, admitted to one of the participating ICUs during the designated study period, and diagnosed with septic shock as defined by the Sepsis-3 criteria. This diagnosis required the presence of hypotension necessitating vasopressor use to maintain a mean arterial pressure of 65 mmHg or higher and a serum lactate level exceeding 2 mmol/L despite adequate fluid resuscitation. Exclusion criteria included patients without complete medical records, specifically those missing baseline SAPS-3 scores or initial lactate levels, and those transferred from other hospitals lacking initial ICU admission data. We also excluded individuals with any directive for terminal care at or before ICU admission, patients under 18 years of age, those in shock due to suicidal actions, and cases with ambiguous shock presentations where sepsis was not clearly the primary cause.

### 2.4. Data Collection

Data extracted from the electronic health records for this study included demographic information such as age, gender, and body mass index (BMI). Clinical metrics recorded upon ICU admission included SAPS-3, and lactate levels noted at initial, peak, and last measurement within the first 24 h of ICU admission, along with the subsequent 24 h lactate clearance. The primary outcome measure assessed was the survival status of patients 28 days after ICU admission.

Lactate clearance was calculated as follows: The difference between the initial lactate level and the lactate level at 24 h was divided by the initial lactate level, then multiplied by 100 to yield a percentage. This metric reflects the percentage decrease in lactate over the first 24 h.

#### Calculation of SAPS-3

A total of 20 characteristics are used for the calculation of SAPS-3, with each expression of one characteristic being assigned with a specific number of points. Each sub-item results in a numeric value, with the sum of the points from all three Boxes being the total SAPS-3, ranging from 0 to a maximum of 217. Before the calculation, each patient receives 16 points for being admitted to the ICU to prevent negative score results. To obtain a better understanding of the components included in SAPS-3 calculation, all 20 characteristics are shown in Table 1 below.

### 2.5. Statistical Methods

Baseline characteristics were summarized using median values with quartiles for continuous variables and proportions for categorical variables. Due to the fact that the majority of the included continuous variables did not follow normal distributions, we opted for this method, which provides a more precise measure of central tendency and variability in such cases and is robust to outliers. Differences between survivors and non-survivors at 28 days were analyzed using the Welch Two-Sample *t*-test for continuous variables and the Chi-squared or Fisher’s exact test for categorical variables.

Logistic regression models were used to determine the odds ratios for 28-day mortality based on SAPS-3 and lactate parameters. Furthermore, multiple logistic regression models were created to describe 28-day mortality using combinations of both SAPS-3 and each of the lactate parameters as independent variables.

The discriminatory power of each predictive model was evaluated by the area under the ROC curve (AUROC). The AUROC values were compared using DeLong’s test, which tests the null hypothesis that two correlated ROC curves are equal, providing insight into whether the integration of lactate measurements with SAPS-3 significantly enhances predictive performance.

All statistical analyses were carried out using the R software package (version 3.6.1, R Foundation for Statistical Computing, Vienna, Austria). Differences were considered statistically significant at a *p*-value of less than 0.05.

## 3. Results

### 3.1. Patient Characteristics

Based on the inclusion and exclusion criteria, 66 patients were identified. The demographic breakdown of these patients showed that a slight majority were male, accounting for 52% of the group. The median age of the participants was 61 years.

In this cohort, the prevalence of septic shock among 826 patients was 8%, with 66 patients experiencing this condition. Of these, 36 (55%) succumbed within the first 28 days following ICU admission or upon diagnosis of septic shock in the ICU, while 30 patients (45%) survived. The population was predominantly male, constituting 52% of the cohort. The mean age of the patients was 59 years (SD ± 15), and the median BMI was 25 kg/m^2^ (23–29).

Blood cultures were positive in 24 of the 66 patients with septic shock, accounting for 36% of this group. Arterial hypertension was the most common comorbidity, present in 42% of the patients, followed by immunosuppression, which was documented in 32% of the cohort, according to the criteria outlined previously [15].

Ventilation and supportive care measures were also notable; 70% of the patients required invasive mechanical ventilation—72% of those who died and 67% of survivors. One-third of the patients underwent hemofiltration therapy during their ICU stay, and 17% received extracorporeal membrane oxygenation (ECMO), of which eight were veno-venous and five were veno-arterial ECMOs, with two patients receiving both sequentially.

Statistically significant differences were observed between the survivors and non-survivors in terms of their SAPS-3, with an average score of 82 (68–90), 86 (75–94) for deceased patients, and 76 (66–86) for survivors (*p* = 0.016). Additionally, the length of ICU stay showed significant variations, with longer stays correlating significantly with a higher probability of survival (*p* < 0.001). Comprehensive baseline characteristics of these patients, including additional demographic and clinical data, are thoroughly detailed in Table 2, divided into two groups according to the primary endpoint of 28-day mortality.

### 3.2. Simplified Acute Physiology Score 3 in Patients with Septic Shock

**Figure 1:**
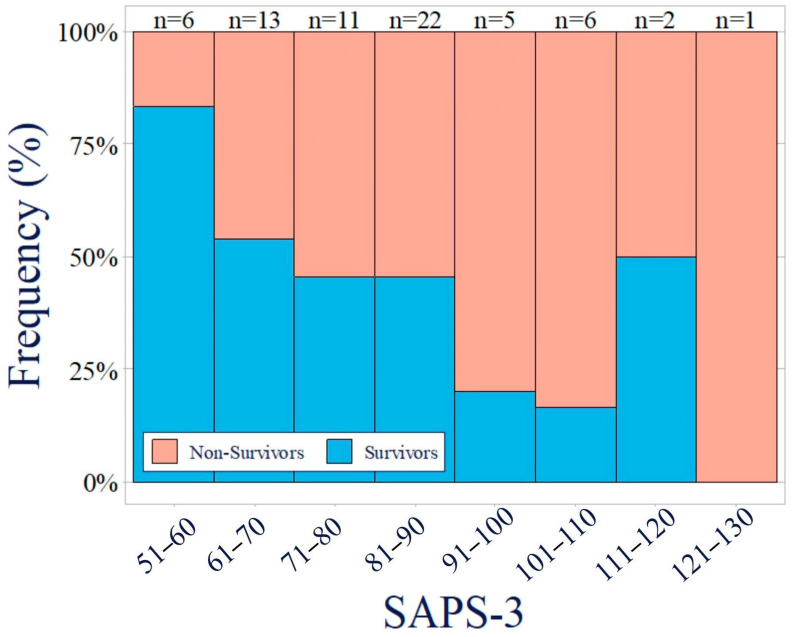
Relative frequencies of SAPS-3 scores in 10-point increments according to 28-day mortality. Survivors are depicted in blue, while non-survivors are shown in red. The data illustrate a significant correlation between SAPS-3 scores and 28-day mortality, as demonstrated by the *t*-test (*p* = 0.016).

The median SAPS-3 in this population was 82 (68–90), ranging from 52 to 122 points. The statistically significant differences in SAPS-3 in relation to 28-day mortality and relative frequencies are illustrated in increments of 10 points (see Figure 1). Of the 14 patients with a SAPS-3 score above 90, only three survived, while eleven passed away. While 56% (17 out of 30) of the surviving patients had a SAPS-3 of 80 or less, only 36.1% (13 out of 36) of the deceased patients had scores within this range.

### 3.3. Lactate Variables in Patients with Septic Shock

The first lactate level measured in the ICU (first lactate) was 2.8 (2.0–4.1) mmol/L among survivors. In contrast, this measure was significantly higher in deceased patients 5.9 (3.9–10.6) mmol/L (*p* < 0.001).

Eight of the 36 deceased patients died within the first 20 h after ICU admission; hence, no measurement of last lactate (inclusion period at 24 ± 4 h from ICU admission) exists for them. Consequently, calculations for peak lactate (for reasons of uniformity, this was defined as the peak value within a time frame of 24 h) and 24 h lactate clearance were also not possible for these patients. Therefore, the sample size was 58 for these three variables.

Measurements of last lactate (next value at 24 h from ICU admission, up to 24 ± 4 h) also significantly differed in terms of 28-day mortality (*p* < 0.001), with a median of 3.6 (2.2–9.8) mmol/L in deceased patients versus 2.1 (1.3–2.8) mmol/L in survivors.

The calculated peak lactate (within 24 ± 4 h) was 6.8 (4.2–13.3) mmol/L in deceased patients and 3.2 (2.6–5.5) mmol/L in survivors. The difference in mean values was 4.5 mmol/L (N = 58) and was statistically significant (*p* < 0.001).

In the analysis, the lactate clearance within the first 24 ± 4 h of treatment (24 h lactate clearance) was calculated, showing no significant difference between deceased and surviving patients (N = 58).

For exploratory purposes, the variables first lactate, last lactate, and peak lactate were log-transformed before they were tested for normal distribution using the Shapiro–Wilk test. Because the null hypothesis (almost) no longer had to be rejected and due to a significant number of ties, *t*-tests were performed afterwards. Conversely, the variable 24 h lactate clearance was analyzed using non-parametric methods due to its distribution characteristics. Table 3 displays the central measures of tendency and variability for these lactate measurements, presenting the actual values without logarithmic transformation.

Among the 41 patients with first lactate levels of up to 5.0 mmol/L, 25 (60.98%) survived the first 28 days. In contrast, among patients with lactate levels greater than 5 and up to 10 mmol/L, only 4 of 14 (28.57%) survived. Of the 11 patients presenting with lactate levels > 10 mmol/L at admission, 10 (90.9%) died, including four within the first 24 h. No survivors were found among those with baseline lactate levels exceeding 15 mmol/L.

The sample size for the analysis of last lactate levels (measured within 24 ± 4 h of admission) included 58 patients, comprising 30 survivors and 28 deceased. A total of 27 out of 44 patients (61%) with septic shock and a last lactate level of up to 5 mmol/L survived the first 28 days, while 17 (38.64%) did not survive. Of the patients who were alive at this point, 14 (24.1%) had a last lactate greater than 5 mmol/L; among these, only 3 (21.43%) survived, all of whom had last lactate levels ≤ 10 mmol/L. All patients in this cohort with a last lactate greater than 10 mmol/L succumbed to their condition. No values over 20 mmol/L were detected at the time of the last lactate measurement.

In comparison to last lactate measurements, only 33 (instead of 44) patients exhibited a peak lactate level ≤ 5 mmol/L. Consequently, 11 of the 44 patients (25%) with a last lactate of ≤5 mmol/L had a peak lactate > 5 mmol/L, indicating a positive clearance between peak and last lactate levels. Among the 14 patients with a peak lactate greater than 5 mmol/L and up to 10 mmol/L, survival was evenly split, with 7 (50%) surviving and 7 (50%) dying within 28 days. Of the 11 patients with a peak lactate greater than 10 mmol/L, 10 (90.9%) subsequently died.

**Figure 2:**
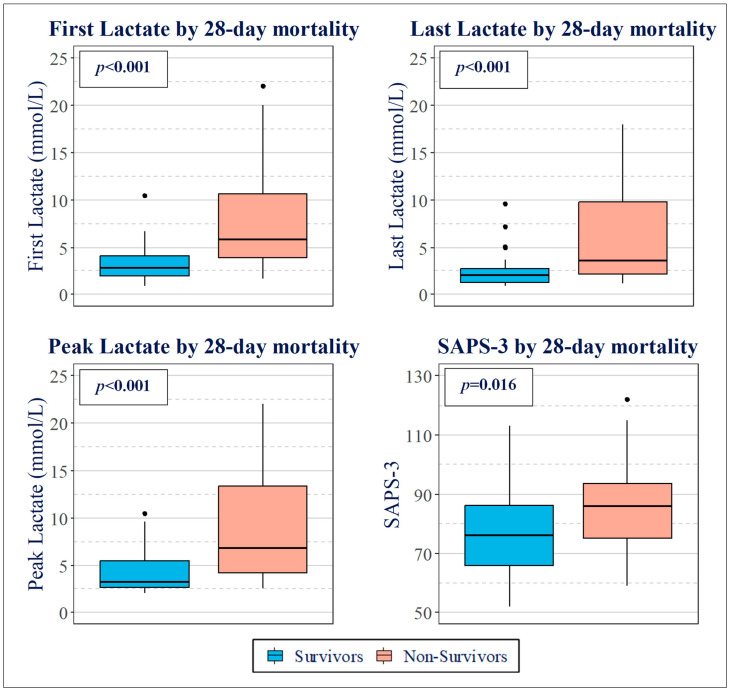
Boxplots of first lactate, last lactate, peak lactate, and SAPS-3 stratified by 28-day mortality. Survivors are depicted in blue and non-survivors in red.

In the first lactate assessment, half of the deceased exhibited levels above 5.9 mmol/L, whereas the vast majority of survivors maintained levels below 4.1 mmol/L. Similar patterns were observed for last lactate and peak lactate levels, with survivors generally presenting lower concentrations. Specifically, for last lactate, survivors predominantly had levels below 2.75 mmol/L, significantly less than the deceased. Peak lactate analyses showed that deceased patients often had levels exceeding 6.8 mmol/L, more than double the median level observed in survivors. Furthermore, SAPS-3 scores correlated with mortality outcomes; survivors typically scored at least 10 points lower than those who did not survive, indicating the score’s relevance in predicting short-term outcomes in septic shock (Figure 2).

### 3.4. Predictive Value of SAPS-3 and Lactate Levels

Simple logistic regression analysis was conducted to assess the impact of several variables, including SAPS-3 and various lactate measurements, on 28-day mortality. Our findings revealed that SAPS-3, first lactate, last lactate, and peak lactate each significantly predicted mortality outcomes (α = 0.05). Of all variables used in the simple logistic regression analysis, the coefficient for first lactate had the highest odds ratio (OR), being 1.511 with a 95% confidence interval (95% CI) of (1.201–2.071). Although the logistic regression analysis showed that an increase in 24 h lactate clearance tended to be associated with a reduction in the odds, this correlation was not significant (*p* = 0.353).

Furthermore, a comprehensive ROC analysis was performed to determine the optimal thresholds for these variables, maximizing the Youden Index and thus identifying cut-off values for clinical applicability. The calculated cut-off values for predicting mortality were 85 points for SAPS-3, 3.6 mmol/L for first lactate, 2.3 mmol/L for last lactate, and 3.2 mmol/L for peak lactate. Additionally, 24 h lactate clearance was analyzed, with a cut-off value determined at 25.72%.

**Figure 3:**
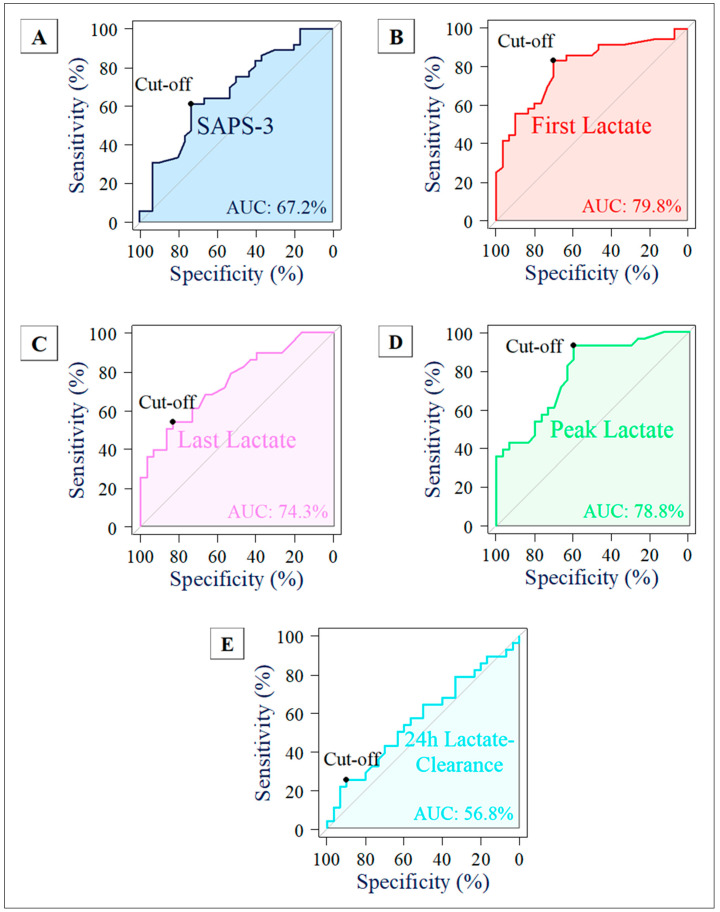
ROC curves for predictive variables in 28-day mortality analysis. (**A**) SAPS-3 (blue), (**B**) first lactate (red), (**C**) last lactate (violet), (**D**) peak lactate (green), and (**E**) 24 h lactate clearance (turquoise). Sample sizes are N = 66 for SAPS-3 and first lactate (**A**,**B**), and N = 58 for the other variables (**C**–**E**).

The discriminative performance of the predictive variables was assessed using receiver operating characteristic (ROC) curves, as illustrated in Figure 3. These figures display the ROC curves for all five variables analyzed in this study. The sample sizes for these analyses were N = 66 for SAPS-3 and first lactate, and N = 58 for the remaining variables. Among the variables, first lactate demonstrated the highest area under the ROC curve (AUROC) at 79.81%, indicating a strong predictive ability regarding 28-day mortality. In contrast, the 24 h lactate clearance exhibited the lowest AUROC, at 56.79%, suggesting a comparatively weaker predictive performance.

### 3.5. Comparative Analysis of AUROC Values

In an exploratory approach, the AUROCs for variables with identical sample sizes were compared using the DeLong test. The difference between the AUROC of SAPS-3 and first lactate, both with a sample size of N = 66, was not statistically significant (79.81% vs. 67.18%, *p* = 0.087). Similarly, for the sample size of N = 58, the difference was also not significant (75.54% vs. 62.44%, *p* = 0.121).

For the comparison of AUROCs among last lactate, peak lactate, and 24 h lactate clearance (N = 58), the significance level was adjusted using the Bonferroni–Holm correction. The difference between the AUROC of last lactate and 24 h lactate clearance was significant (74.29% vs. 56.79%, *p* = 0.009). Furthermore, the difference between peak lactate and 24 h lactate clearance was also significant (78.81% vs. 56.79%, *p* = 0.034). However, the difference between last and peak lactate was not significant (*p* = 0.432).

### 3.6. Comparative Analysis of Lactate Measures and SAPS-3

In the stepwise “backwards” selection process, the best model for describing outcomes excluded SAPS-3, favoring a combination of first lactate and last lactate instead. Notably, 24 h lactate clearance was not included in this analysis due to its functional relationship with first and last lactate.

Subsequent models were developed with 28-day mortality as the dependent variable, incorporating SAPS-3 and one of the lactate variables as independent predictors. Initially, the model including SAPS-3 and last lactate was computed, where only the coefficient for last lactate (0.283) was statistically significant, with a standard error of 0.111 and a Z-value of 2.545 (N = 58, *p* = 0.011).

Further analysis involved multiple logistic regressions using SAPS-3 and first lactate (N = 66). The results indicate that the coefficient for first lactate and the model’s intercept were statistically significant, whereas the coefficient for SAPS-3 is not. In this regression model, the coefficient for first lactate was 0.385 with a standard error of 0.141 and a Z-value of 2.725 (*p* = 0.006).

Additionally, a multiple logistic regression was calculated for SAPS-3 and peak lactate. This model demonstrated that only the coefficient for peak lactate was statistically significant (N = 58), with a coefficient of 0.289 and an associated standard error of 0.109 and a Z-value of 2.863 (*p* = 0.004).

### 3.7. Evaluating the Predictive Accuracy of SAPS-3 and Lactate Combinations Using ROC Analysis

ROC curves were then computed for each of these three regression models to compare their AUROCs with that of SAPS-3 from a simple logistic regression using the DeLong test. This targeted analysis did not adjust the significance level, maintaining α = 0.05, due to the predefined number of tests.

**Figure 4:**
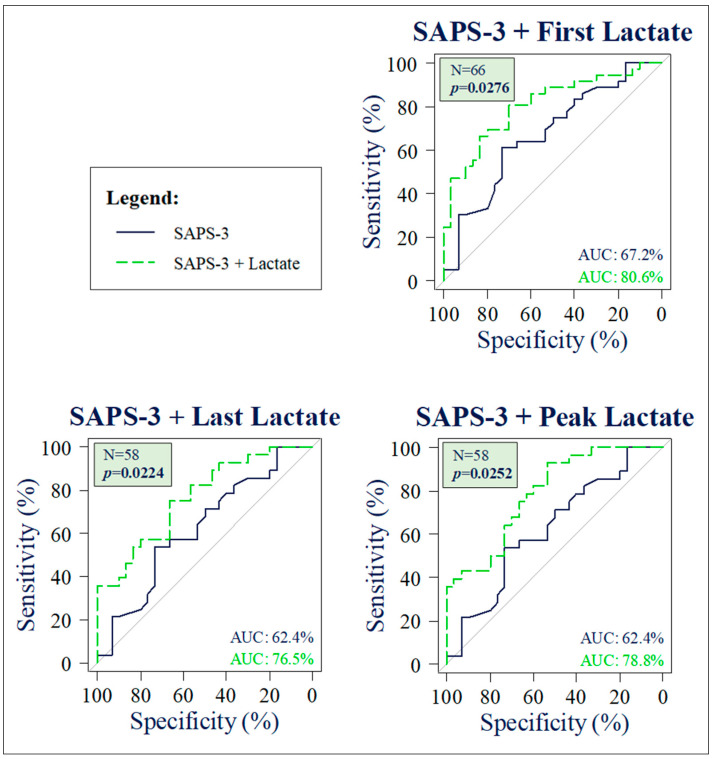
Comparative ROC curves for prognostic models in septic shock. ROC curves for SAPS-3 alone are depicted in dark blue, and those for SAPS-3 in combination with lactate are depicted in green.

The AUROC of the ROC curve from the regression model incorporating the independent variables SAPS-3 and first lactate was 80.56% for the entire population (N = 66). The result of the DeLong test, which compared this AUROC with that of SAPS-3 alone, was statistically significant (67.18% vs. 80.56%, *p* = 0.028). These two ROC curves are compared in Figure 4.

The optimal cut-off value for this population was a SAPS-3 score of 79.76 combined with a first lactate level of 3.68 mmol/L, achieving a sensitivity of 80.56% and specificity of 70.0%.

Comparison of the AUROC from the simple logistic regression model (SAPS-3, N = 58) with that from the multiple logistic regression model for the independent variables SAPS-3 and last lactate using DeLong tests was statistically significant (76.55% vs. 62.44%, *p* = 0.022). This comparison is also illustrated in Figure 4.

The optimal cut-off value for this population was a SAPS-3 of 75.69 combined with a last lactate level of 2.2 mmol/L, yielding a sensitivity of 75.0% and specificity of 66.67%.

The result of the DeLong test comparing the AUROC of the model SAPS-3 + peak lactate with the AUROC of the regression model for SAPS-3 alone (N = 58) was statistically significant (78.81% vs. 62.44%, *p* = 0.0252). The two corresponding ROC curves are compared in Figure 4. The optimal cut-off value for this population was a SAPS-3 score of 69.16 combined with a peak lactate level of 3.2 mmol/L, resulting in a sensitivity of 92.86% and specificity of 53.33%.

## 4. Discussion

The management of sepsis and septic shock remains a significant challenge for clinical caregivers. This study aimed to investigate the predictive utility of two key markers, SAPS-3 and lactate levels, in patients with septic shock. Despite the widespread use of SAPS-3 and point-of-care lactate measurements in intensive care units, the combined assessment of these two markers has not been extensively studied. Our results have provided valuable insights into improving clinical decision-making and management for these critically ill patients.

The study highlighted the high mortality rate of 55% in this population despite treatment in a tertiary care center of excellence, emphasizing the need for continuous research to improve patient outcomes. While deceased and surviving patients shared similar baseline characteristics, they differed significantly in prognostic markers like SAPS-3 (*p* = 0.016) and lactate (*p* < 0.001 for first, last, and peak lactate). Recent studies have reported similar findings, but there is still a lack of comparable data due to evolving sepsis definitions, leading to reduced septic shock incidence. Studies often include broader sepsis populations, not just those in shock [16].

The mortality rate for septic shock in this study was high, especially compared to recent mixed severe sepsis and septic shock cohorts, where the rates ranged from 24 to 35% [17,18,19]. Even in some pure shock cohorts, the mortality rate was lower, such as 24% [20]. Nevertheless, the high mortality observed in our study aligns with previous observations that septic shock mortality typically ranges from 40% to 60% [21,22,23], reaching as high as 80% in the control group of the seminal esmolol trial [24].

A significant factor contributing to this high mortality rate was the severity of illness, reflected in the elevated SAPS-3. This study’s median SAPS-3 was 82, whereas a previous study investigating a mortality prediction model for sepsis patients recorded a lower SAPS-3 of 65 (57–75), with a corresponding 25.3% mortality rate [25]. Additionally, there was a high prevalence of active cancer in our cohort due to the hematooncologic affiliation of one of the participating ICUs, with 36% of patients affected and thus far exceeding the 15% reported previously [26]. Furthermore, there was a high proportion of immunosuppressed patients (32%) in this cohort, which was notably higher than the published rates of 13.7% to 24.1% [27].

In total, 65% of our patients had culprit pathogens identified due to rigorous assessments and timed specimen collection. This aligns well with previous trial populations where up to 30% of sepsis cases remained unidentified [17], while other studies showed that 28–89% of sepsis cases had no identifiable pathogen by culture [28,29,30].

Our study showed that first lactate < 4 mmol/L is a positive sign if it does not increase beyond that level. However, a first lactate > 10 mmol/L is a very critical threshold, and those who could not decrease this level within 24 h had universally fatal outcomes. If first lactate even exceeded 15 mmol/L, prognosis was essentially grim. Accurate prediction of survival is essential for developing effective therapeutic strategies and making informed clinical judgments about prognosis. This information is invaluable for ICU clinicians, as communicating prognosis to family members plays a central role in their care responsibilities, helping families to prepare for potential outcomes and understand treatment plans [31]. Moreover, understanding the likely prognosis allows clinicians to avoid interventions that are unlikely to be beneficial and could diminish the quality of life for patients, families, and caregivers. This ensures that remaining time is spent meaningfully, providing emotional support and care tailored to the patient’s needs [32].

All lactate measurements were better predictors than SAPS-3 alone, except for lactate clearance, which was not predictive, likely because survivors had low initial lactate levels and thus little potential for clearance, whereas those with high lactate often died early. Previous studies [10,33,34,35] had demonstrated lactate clearance to be a predictive marker; however, these studies had outdated definitions and included non-shock sepsis patients.

Combining SAPS-3 with other biomarkers, such as IL-6 and lactate, as demonstrated previously [36,37], could significantly enhance the predictive accuracy of patient outcomes. In our study, first lactate proved to be the most effective predictor of outcomes when combined with SAPS-3, with an AUC of 80.6%. This finding aligns with Nguyen et al. [10], who highlighted the importance of early lactate prediction due to its strong implications for patient survival. However, it is worth noting that first lactate had a more substantial dataset available compared to last lactate, as patient deaths before the 24 h mark resulted in fewer last lactate measurements being recorded.

In our previous study on cardiogenic shock, last lactate proved to be the most predictive marker, likely due to the potential for myocardial recovery [38]. However, in the present study focusing on septic shock, first lactate emerged as the more crucial predictor. This distinction might be attributed to the severity of the initial insult in septic shock, which often triggers a downward spiral of systemic inflammation and vasoplegia that is likely irreversible beyond a certain point, significantly reducing the patient’s ability to recover [39]. Therefore, the early identification of elevated lactate levels in septic shock is critical for assessing patient outcomes, emphasizing the importance of first lactate as a prognostic marker in this context.

A new predictive marker combining SAPS-3 and lactate could offer a promising approach, although further large-scale prospective studies are necessary.

### Limitations, Strengths, and Directions for Future Research

This study presents several limitations that may affect the generalizability of our findings. The primary constraints include its retrospective design, small sample size, and the fact that it was conducted at a single center. These factors might limit the broader applicability of the results. Additionally, the robustness of our conclusions is impacted by limitations in documentation, such as inconsistent parameters and instances of missing data. The diversity and size of the patient cohort, variations in pre-ICU care, and potential biases introduced during lactate measurement due to medical interventions further influence the generalizability of our findings. Despite these challenges, the strength of this study lies in its thorough review of documentation and expert resolution of diagnostic uncertainties, which ensures a comprehensive and accurate inclusion of cases of septic shock. Future research should focus on validating the integration of SAPS-3 and lactate measurements across larger, more diverse populations to confirm the findings of this study. Additionally, exploring the inclusion of other biomarkers alongside SAPS-3 may further enhance prognostic accuracy. Longitudinal studies could also be beneficial to tracking long-term outcomes in septic shock survivors, providing insights into the efficacy of immediate interventions and the potential benefits of real-time biomarker monitoring in ICU settings. These studies will help refine treatment protocols and improve patient outcomes in critical care.

## 5. Conclusions

Integrating SAPS-3 with lactate measurements, particularly first lactate, improves predictive accuracy for 28-day mortality in septic shock patients. First lactate serves as an early, robust prognostic marker, providing crucial information for clinical decision-making and care prioritization. Further large-scale studies are needed to refine these predictive tools and validate their efficacy in guiding treatment strategies.

## Figures and Tables

**Table 1:** The Simplified Acute Physiology Score 3 (SAPS-3).

Characteristic	Expressions	Points
Box I		
○ Age	<40 years	0
	≥40 and <60 years	5
	≥60 and <70 years	9
	≥70 and <75 years	13
	≥75 and <80 years	15
	≥80 years	18
○ Pre-existing co-morbidities	Cancer therapy	3
	Chronic heart failure (NYHA IV), hematological cancer	6
	Cirrhosis, AIDS * * double points if the patient has both conditions	8 (16)
	Cancer	11
○ Use of vasoactive drugs before ICU admission	No	0
	Yes	3
○ Length of stay in the hospital before ICU admission (days)	<14 days	0
	≥14 and <28 days	6
	≥28 days	7
○ Location before ICU admission (intra-hospital)	Emergency room	5
	Other ICU	7
	Other	8
Box II		
○ ICU admission was unplanned	No	0
	Yes	3
○ Reason(s) for ICU amission	Rhythm disturbances (cardiovascular); * See “Seizures” below	−5
	Seizures (neurologic); * If the patient is also admitted due to rhythm disturbances, only the worse value (−4) is added	−4
	Hypovolemic hemorrhagic shock, hypovolemic non-hemorrhagic shock (cardiovascular)/acute abdomen, other (digestive); * Double points if the patient has both conditions	3 (6)
	Coma, stupor, obtuned patient, vigilance disturbances, confusion, agitation, delirium (neurologic)	4
	Septic shock, anaphylactic shock, mixed and undefined shock (cardiovascular)	5
	Liver failure (hepatic)	6
	Focal neurologic deficit (neurologic)	7
	Severe pancreatitis (digestive)	9
	Intracranial mass effect (neurologic)	10
	All others	0
○ Surgery before ICU admission	Scheduled surgery	0
	No surgery	5
	Emergency surgery	6
○ Anatomical site of surgery	Transplantation surgery (liver, kidney, pancreas, kidney and pancreas, other)	−11
	Trauma—other, isolated: (incl. thorax, abdomen, limb) and trauma—multiple	−8
	Cardiac surgery (CABG without valvular repair)	−6
	Cerebrovascular accident (neurosurgery)	5
	All others	0
○ Presence of infection at ICU admission (none/nosocomial/respiratory)	No	0
	Nosocomial	4
	Respiratory	5
Box III		
○ Highest total bilirubine level (mg/dL)	<2	0
	≥2 and <6	4
	≥6	5
○ Highest body temperature (°C)	<35	7
	≥35	0
○ Highest creatinine level (mg/dL)	<1.2	0
	≥1.2 and <2	2
	≥2 and <3.5	7
	≥3.5	8
○ Highest heart rate (beats per minute)	<120	0
	≥120 and <160	5
	≥160	7
○ Highest leukocyte level (G/L)	<15	0
	≥15	2
○ Lowest hydrogen ion concentration (pH)	≤7.25	3
	>7.25	0
○ Lowest platelet count (G/L)	<20	13
	≥20 and <50	8
	≥50 and <100	5
	≥100	0
○ Lowest systolic blood pressure (mmHg)	<40	11
	≥40 and <70	8
	≥70 and <120	3
	≥120	0
○ Lowest estimated Glasgow Coma Scale (points)	3–4	15
	5	10
	6	7
	7–12	2
	≥13	0
○ Oxygenation index and ventilatory support (FiO_2_, mechanical ventilation y/n)	PaO_2_/FiO_2_ < 100 and mechanical ventilation	11
	PaO_2_/FiO_2_ ≥ 100 and mechanical ventilation	7
	PaO_2_ < 60 and no mechanical ventilation	5
	PaO_2_ ≥ 60 and no mechanical ventilation	0

Abbrevations: ICU: Intensive Care Unit; NYHA: New York Heart Association; CABG: Coronary Artery Bypass Graft; PaO_2_: Arterial Partial Pressure of Oxygen; FiO_2_: Fraction of Inspired Oxygen.

**Table 2:** Baseline parameters.

Characteristic	N	Overall ^1^	28-Day Mortality		*p*-Value ^2^
			Non-Survivors, N = 36 ^1^	Survivors, N = 30 ^1^	
Demographic Baseline Parameters					
Age (years)	66	61 (48–71)	67 (56–72)	55 (43–65)	0.021
Sex	66				0.5
Male		34/66 (52%)	20/36 (56%)	14/30 (47%)	
Female		32/66 (48%)	16/36 (44%)	16/30 (53%)	
BMI ***^a^*** (kg/m^2^)	66	26 (23–29)	26 (23–30)	25 (23–28)	0.3
Severity of Illness Score					
SAPS-3	66	82 (68–90)	86 (75–94)	76 (66–86)	0.016
Information concerning the ICU stay					
ICU length of stay (days)	66	4 (1–20)	2 (1–4)	15 (6–35)	<0.001
Transfer from external hospital	66	11/66 (17%)	6/36 (17%)	5/30 (17%)	>0.9
ICU readmission	66	1/66 (1.5%)	1/36 (2.8%)	0/30 (0%)	>0.9
Post-operative ICU admission	66	4/66 (6.1%)	1/36 (2.8%)	3/30 (10%)	0.3
Surgery during ICU stay	66	7/66 (11%)	1/36 (2.8%)	6/30 (20%)	0.041
Pre-existing conditions					
Hypertension	66	28/66 (42%)	16/36 (44%)	12/30 (40%)	0.7
Diabetes mellitus (DM)	66	11/66 (17%)	5/36 (14%)	6/30 (20%)	0.5
COPD	66	13/66 (20%)	8/36 (22%)	5/30 (17%)	0.6
Malignancy (active cancer)	66	13/66 (20%)	7/36 (19%)	6/30 (20%)	>0.9
Coronary artery disease	66	8/66 (12%)	4/36 (11%)	4/30 (13%)	>0.9
Peripheral artery disease (including cerebrovascular)	66	5/66 (7.6%)	3/36 (8.3%)	2/30 (6.7%)	>0.9
Chronic renal failure	66	9/66 (14%)	5/36 (14%)	4/30 (13%)	>0.9
Immunosuppression	66	21/66 (32%)	13/36 (36%)	8/30 (27%)	0.4
Pathogen identified					
Overall	66	43/66 (65%)	24/36 (67%)	19/30 (63%)	0.8
Positive blood culture	66	24/66 (36%)	14/36 (39%)	10/30 (33%)	0.6
Detection of pathogens in tracheal secretions/BAL	66	18/66 (27%)	9/36 (25%)	9/30 (30%)	0.6
Positive urine culture test	66	17/66 (26%)	9/36 (25%)	8/30 (27%)	0.9
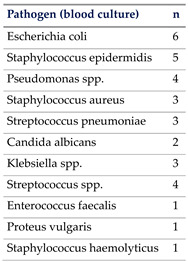		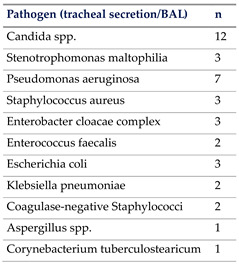		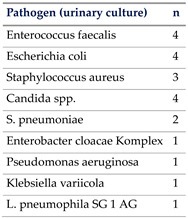 * In 2 patients, respiratory pathogens (*Legionella* and *S. pneumoniae*) were detected via urinary antigen tests	
Intensive care treatment					
ECMO therapy	66	11/66 (17%)	5/36 (14%)	6/30 (20%)	0.5
Hemofiltration	66	22/66 (33%)	12/36 (33%)	10/30 (33%)	>0.9
Invasive ventilation	66	46/66 (70%)	26/36 (72%)	20/30 (67%)	0.6
Antibiotic treatment	66	65/66 (98%)	35/36 (97%)	30/30 (100%)	>0.9
*Meropenem*	66	28/66 (42%)	14/36 (39%)	14/30 (47%)	0.5
*Piperacillin*/*Tazobactam*	66	36/66 (55%)	23/36 (64%)	13/30 (43%)	0.095
*Ampicillin*/*Sulbactam*	66	1/66 (1.5%)	1/36 (2.8%)	0/30 (0%)	>0.9
*Linezolid*	66	13/66 (20%)	5/36 (14%)	8/30 (27%)	0.2
Others	66	52/66 (79%)	25/36 (69%)	27/30 (90%)	0.042
Antifungal treatment	66	19/66 (29%)	9/36 (25%)	10/30 (33%)	0.5
*Fluconazole*	66	8/66 (12%)	4/36 (11%)	4/30 (13%)	>0.9
*Voriconazole*	66	1/66 (1.5%)	1/36 (2.8%)	0/30 (0%)	>0.9
*Caspofungin*	66	1/66 (1.5%)	0/36 (0%)	1/30 (3.3%)	0.5
*Micafungin*	66	10/66 (15%)	4/36 (11%)	6/30 (20%)	0.5
Others	66	5/66 (7.6%)	0/36 (0%)	5/30 (17%)	0.016
IV ***^b^*** vasoactive drugs	66	65/66 (98%)	35/36 (97%)	30/30 (100%)	>0.9
*Norepinephrine*	66	64/66 (97%)	35/36 (97%)	29/30 (97%)	>0.9
*Dobutamine*	66	30/66 (45%)	20/36 (56%)	10/30 (33%)	0.071
*Vasopressin*	66	28/66 (42%)	20/36 (56%)	8/30 (27%)	0.018
*Hydrocortisone*	66	33/66 (50%)	19/36 (53%)	14/30 (47%)	0.6
CPR ***^c^*** prior to admission	66	5/66 (7.6%)	5/36 (14%)	0/30 (0%)	0.058

**^1^** Median (quartiles) or n/N (%); **^2^** Welch Two-Sample *t*-test; Pearson’s Chi-squared test; Fisher’s exact test; ***^a^*** Body mass index; ***^b^*** Intravenous; ***^c^*** Cardiopulmonary resuscitation.

**Table 3:** Lactate measurements.

Characteristic	N	Overall ^1^	28-Day Mortality		*p*-Value ^2^
			Non-Survivors, N = 36 ^1^	Survivors, N = 30 ^1^	
First lactate (mmol/L)	66	4.1 (2.5–6.7)	5.9 (3.9–10.6)	2.8 (2.0–4.1)	<0.001
Last lactate (mmol/L)	58	2.5 (1.7–5.0)	3.6 (2.2–9.8)	2.1 (1.3–2.8)	<0.001
Peak lactate (mmol/L)	58	4.4 (3.0–8.1)	6.8 (4.2–13.3)	3.2 (2.6–5.5)	<0.001
24 h lactate clearance (%)	58	24.9 (−25.0–47.9)	19.9 (−34.4–41.4)	26.7 (−16.5–49.5)	0.4

**^1^** Median (quartiles). **^2^** Welch Two-Sample *t*-test; Wilcoxon rank sum test.

## Data Availability

The data that support the findings of this study are not openly available due to reasons of sensitivity but are available from the corresponding author upon reasonable request.
